# An Exploration of the Goodness of Fit of Web-Based Tools for Māori: Qualitative Study Using Interviews and Focus Groups

**DOI:** 10.2196/50385

**Published:** 2024-05-02

**Authors:** Liesje Donkin, Marie-Claire Bidois-Putt, Holly Wilson, Penelope Hayward, Amy Hai Yan Chan

**Affiliations:** 1 Department of Psychology and Neuroscience Auckland University of Technology Auckland New Zealand; 2 School of Pharmacy The University of Auckland Auckland New Zealand

**Keywords:** Indigenous people, Māori, eHealth, mental health, web-based intervention, digital intervention

## Abstract

**Background:**

Indigenous communities often have poorer health outcomes and services under traditional models of care. In New Zealand, this holds true for Māori people who are tāngata whenua (the indigenous people). Several barriers exist that decrease the likelihood of indigenous communities often have poorer health outcomes and poor service fit under traditional models of care, including access issues, systemic and provider racism, and a lack of culturally safe and responsive services. Web-based interventions (WBIs) have been shown to be effective in supporting mental health and well-being and can overcome some of these barriers. Despite the large number of WBIs developed, more investigation is needed to know how well WBIs fit with an indigenous worldview and how they meet the needs of indigenous communities so that a digitally based future does not drive social and health inequities.

**Objective:**

This study aims to explore the goodness-of-fit of WBIs of Māori individuals, the indigenous people of Aotearoa/New Zealand.

**Methods:**

We used interviews (n=3) and focus groups (n=5) with 30 Māori participants to explore their views about WBIs. Interviews were analyzed using reflexive thematic analysis by members of the research team.

**Results:**

Overall, there was a perception that the design of WBIs did not align with the Māori worldview, which centers around people, relationships, spirituality, and holistic views of well-being. A total of 4 key themes and several subthemes emerged, indicating that WBIs were generally considered a poor fit for Māori. Specifically, the themes were as follows: (1) WBIs are disconnected from the core values of te ao Māori (the Māori worldview), (2) WBIs could be helpful in the right context, (3) there are significant barriers that may make it harder for Māori to use WBIs than other groups, and (4) ways to improve WBIs to help engagement with Māori.

**Conclusions:**

While WBIs are often considered a way to reduce barriers to care, they may not meet the needs of Māori when used as a stand-alone intervention. If WBIs are continued to be offered, developers and researchers need to consider how to develop WBIs that are responsive and engaging to the needs of indigenous communities rather than driving inequities. Ideally, WBIs should be developed by the people they are intended for to fit with those populations’ world views.

## Introduction

### Background

Web-based interventions (WBIs) are therapeutic interventions delivered over technological means such as mobile phones or the internet to facilitate behavior change to improve health or well-being. Over the past 2 decades, the explosion in the number of WBIs has paralleled the growth and increased access to technology worldwide. This growth is driven by the potential of WBIs to reach more people at a lower cost than comparable face-to-face therapies. Several reviews now report that WBIs are as effective as face-to-face interventions [1-4], making WBIs a viable alternative to conventional therapies.

Indigenous communities often face more significant barriers to accessing health care than the dominant cultural group [5-9]. The barriers include systemic racism [10-14], negative attitudes held by health care professionals about indigenous people and traditional methods of healing [14-17], a lack of culturally responsive and safe services [18-20], and significant psychosocial barriers that make even getting to appointments difficult [21]. Due to these barriers and the social inequities created by colonization [22-24], indigenous people often experience worse health and social, psychological, and mortality outcomes. Given the potential benefits of WBIs to overcome some of these barriers, WBIs are increasingly being developed for the *hard to reach or engage groups* [25-27], such as indigenous communities.

However, one of the reasons that traditional health care underserves indigenous communities is that the care delivery model is often driven by a Western or dominant cultural paradigm [17], which often does not fit with the values and worldview of the indigenous community [28,29]. Thus, existing Western and biomedical models may further perpetuate health inequities [30]. For example, many indigenous communities such as Māori (the indigenous people of New Zealand) [31] hold a collectivist worldview rather than the individualized view prevalent in Western medicine [31,32]. In te ao Māori (the Māori worldview), the relationship and generational history between 2 people are paramount and influence engagement with others. Similarly, wairua (spirituality), Mauri (life force), whakapapa (history), and tikanga (ways of doing things) are paramount in all interactions. These factors are often deprioritized in the medical model, where an adequate clinician-patient relationship is assumed. Similarly, there is often a belief that this relationship will progress within what is largely superficial, impersonal and not responsive to the patient’s cultural needs nor recognizes the history and impact of colonization [33].

As the uptake and persistence of WBIs are often considered low, particularly on a long-term basis [34,35], it is essential to understand the views of potential users to inform the design and determine if WBIs are the best form of intervention. This has been frequently explored with nonindigenous populations, which found that time constraints and lack of perceived worth of the WBI were key reasons for stopping using WBIs [35]. In indigenous communities, uptake of WBIs has been found to be variable and can be affected by intervention characteristics [36,37]. In addition to this, many indigenous communities have poor access to technology and technological infrastructure [38], which can create further barriers to accessing WBIs. Understanding barriers to uptake can help researchers, developers, and policy makers consider how to improve WBIs for indigenous populations or if WBIs are even an appropriate intervention strategy for indigenous communities—because without this understanding, investment in technology may further drive health inequities.

### Objective

Currently, there is limited research exploring the views of Māori adults about WBIs and the fit of WBIs with te ao Māori. Given this, this study sought to understand the views of Māori adults about WBIs using a qualitative design and reflexive thematic analysis of interviews and focus groups.

## Methods

### Overview

This study used a qualitative methodology using a mix of web-based videoconferencing interviews, face-to-face interviews, and focus groups. All interviews were facilitated by MCBP, who also recruited participants using snowball recruitment and social media advertising.

### Participants

People were recruited into the study if they identified as Māori, were aged >18 years, and could consent to participation either orally or in a written format. There were no exclusion criteria for this study.

### Recruitment

Participants were recruited through a range of recruitment methods using convenience sampling. Participants for 1 focus group (n=8) were recruited through Te Kete Pounamu, a nationally based organization for Māori with lived experience of mental distress and addiction. The remainder of the participants were recruited via the researchers’ professional and personal networks, web-based advertisements, and through relationships formed in the community by research team members.

### Procedure

The participants were offered a choice of participating in an individual interview or a focus group discussion. During the initial stages of the meeting with the participants, the interviewer (MBP) opened the session with karakia (prayer) if the participants wished for this to happen. The interviewer then engaged with participants in whanaungatanga (building relationships through shared connection). The participants were then reoriented to the study, and consent was obtained in a written or recorded verbal format. The participants then confirmed that they were happy to be recorded, and the interview or focus group commenced.

Interviews initially started by providing the participants with a definition of WBIs and then asking participants about their views of WBIs. If needed, the participants were prompted to talk about why they felt WBIs did or did not fit with their worldview. A specific example of an existing WBI was used as a talking stimulus by demonstrating the WBI if required. At the end of the session, participants were thanked for their time, and the session was closed with karakia if it was opened with one.

### Data Gathering

Given that Māori have a strong oral history and prefer to engage kanohi-ki-te-kanohi (face-to-face), interviews and focus groups were used. Combining both methods meant that participants could be part of a group to share their views or talk individually. Consultation with Te Kete Pounamu indicated that their tāngata whaiora (people seeing health) would likely prefer to engage in a focus group. Thus, focus groups were offered to allow this. Individual interviews were offered to enable flexibility in interview times at a time and place that suited individuals. Group interviews were complete kanohi-ki-te-kanoi, while individual interviews were a mix of kanohi-ki-te-kanohi and on the web. Both interviews and group interviews used the same semistructured interview guide with questions to facilitate reflective discussion. Examples of questions used were as follows: “What are your views on digital interventions to support mental health and well-being?” “What do you think has led to you developing these views?” “How does the use of digital interventions fit your background and culture?”

A total of 8 transcripts were produced, consisting of 3 individual interviews and 5 interviews with >2 participants. A total of 30 people were interviewed as part of the study. The interviews were manually transcribed (by HW); checked (by LD); and coded using inductive reflexive thematic analysis by Clarke et al [39] by 2 members (LD and MCBP) of the research team individually and following a 6-step process of familiarization, coding, generating themes, reviewing themes, defining and naming themes, and writing up. Reflexive thematic analysis was chosen as an appropriate methodology for this study as it is aligned with recent projects seeking to understand the views of Māori [40-43] as tāngata whaiora (those seeking health).

The transcripts were not returned to the participants for review. Any disagreements in coding between LD and MCBP were resolved by discussion between LD, MCBP, and PH. Codes, subthemes, and themes were combined on the web for review by the rest of the research team using a visual collaboration platform [44]. The wider team (LD, MCBP, HW, PH, and AHYC) further discussed the codes and themes.

### Ethical Considerations

Ethics approval for the study was received from the Auckland Health Research Ethics Committee (AHREC AH23110; expiry October 18, 2024).

Written consent was provided by all participants who completed face-to-face interviews or focus groups following a review of the participant information sheet. In web-based interviews, an oral consent protocol was followed where participants were video recorded giving their consent. The researchers then completed the consent forms based on participant responses, and consents were electronically provided to the participants. The participants had the option to provide scanned copies of the signed consent form to the interviewer if they preferred this method over video consent.

One participant completed their interview or focus group, the recordings were transcribed (completed by MCBP or HW), and all identifying information was removed from the transcript. The deidentified transcripts were provided to the wider research team for review. LD led the review process with MCBP. Codes and themes were discussed with HW, AHYC, and PH once coding was complete. The original recordings were stored separately from the transcripts in a secure manner on a password-protected university-managed research drive aligned with the data management plan.

During the research process, Koha (a gift acknowledging the time spent by participants and sharing their knowledge) in the form of NZ $20 (US $11.87) supermarket vouchers and kai (food) were provided to participants.

## Results

### Participants

A total of 30 participants participated in this research across 5 group interviews and 3 individual interviews (Table 1).

In terms of participants, ages ranged from 18 to 74 years, with the mean participant age being 41.3 (SD 19.6) years. Of the 30 participants, 11 (37%) identified as a man, 12 (40%) as a woman, 6 (20%) did not specify their gender, and 1 (3%) identified as nonbinary. The participants lived in a mix of main urban centers, such as Auckland and Hamilton, and in more urban areas, such as Northland and Rotorua.

**Table 1:** Breakdown of the research participation by data collection mode (N=30).

Transcript number	Format	Label	Participants, n (%)	Duration (minutes)
1	Group	Group 1	12 (40)	184
2	Individual	Interview 1	1 (3)	49
3	Group	Group 2	8 (27)	180
4	Group	Group 3	3 (10)	57
5	Individual	Interview 2	1 (3)	56
6	Individual	Interview 3	1 (3)	53
7	Group	Group 4	2 (7)	65
8	Group	Group 5	2 (7)	68

### Findings

The participants were largely aligned with their views on WBIs. Although they often offered a critical perspective on WBIs, they also quickly indicated that others might find WBIs helpful even though they did not believe that WBIs would be useful for themselves. Through the analysis of the transcripts, 4 themes emerged, as listed in Table 2.

These themes and subthemes are discussed in more detail in the subsequent sections.

**Table 2:** Themes and subthemes generated from the data.

Theme number	Theme	Subtheme
1	WBIs^a^ are disconnected from the core values of te ao Māori (the Māori worldview)	Te ao Māori is about wairua, and WBIs cannot replicate this. Māori models of health are holistic, whereas WBIs are singular in focus. WBIs could be a strand in the weaving of a well-being kete (basket).
2	WBIs could be helpful in the right context	WBIs could be useful for people who “moved with the times.” Apps could be a tool but not the solution—a blended care approach is needed.
3	Barriers to using WBIs for Māori	WBIs come with an upfront cost that may drive inequitable access. Technical issues put people off. Literacy and language may make engagement difficult. Mistrust due to years of systemic racism and broken promises.
4	Ways to improve WBIs to help engagement with Māori	Māori imagery is key for Māori to connect. Māori models of well-being should be at the heart of all interventions. Improved integration of the te reo Māori language would make WBIs more appealing. By Māori for Māori.

^a^WBI: web-based intervention.

### WBIs Are Disconnected From the Core Values of Te Ao Māori

#### Overview

Te ao Māori (the world view of Māori) and Māori culture are built around relationships, collectivism, and a shared connection. Considering the collective is vital, and a treatment model focused on the individual’s pathology alone without connection or relationships is at odds with Māori beliefs and values. The participants felt that WBIs may not understand and replicate the critical relational aspect, which is crucial in all aspects of life for Māori. Therefore, most participants did not consider WBIs as something that would fit with Māori culture or worldview. A participant said the following:

Because you are not face to face...and us Māori’s, we are...like, unless we know you and love you, we’ll never be as open though. No way! Ah, it’s, it’s, I don’t know...it just is

Group 5

Another participant stated the following:

I definitely think doing something online definitely lacks whanaungatanga [relationships/the process of forming relationships] and that kind of personal relationships with your doctor or your medical provider

Group 5

Many of the older participants struggled to conceptualize what an automated program such as a chatbot would look like and how this could be used to support well-being, as this was removed from the concept of relationships, which are key to wellness and healing. Even when considering web-based therapy delivery, this had significant barriers to developing whanaungatanga. For some, there was a perception that without a real in-person connection with others, it would be easy to mask true feelings and intentions, which would further limit the benefits of the WBI. Others felt that people might inadvertently disclose more than they wanted to due to the WBI not seeming like a relationship (and more like a diary), and this could be harmful due to mistrust in how this information would be used. The overall message is that a person’s absence meant the absence of connection and healing. A participant said the following:

For healing purposes, people are necessary in our culture

Group 1

Another participant stated the following:

I know people, they would like the online thing because they don’t feel like they are disclosing much about themselves. They are, but they don’t feel like they are

Interview 2

Another participant said the following:

At the same time, when its online you can kind of feel some like you’re wearing a mask. So, it’s not really you

Interview 3

The perceived lack of connection for users with WBIs hindered honesty and information sharing. This ultimately limited the benefits and healing that could be obtained when exclusively using WBIs. For some participants, there was the belief that WBIs and the digital world contributed to poor health as people became more disconnected from others; lost their ability to communicate; and tended to live in a digital world that was disconnected from their whakapapa (family history or genealogy), whenua (land), atua (gods or spiritual beings), and values. Some perceived turning to a digital world for help as a lack of personal responsibility for healing, which would further exacerbate long-term difficulties and have implications for future generations. This was particularly emphasized for automated interventions with a lack of relationship and accountability. A participant stated the following:

I do I think there is a whole generation, like the younger generation, who will know nothing else but digital stuff and like babies who now are growing up seeing nothing but masks on people’s faces. It’s the same kind of thing they won’t know how to relate unless it’s seen, or read it, or hear it. You know, tap into it online. You know? Their connections might be doing the de-de-de finger scrolling online, whatever online that might be their connection. But for me, I’m old school. I like people

Group 1

#### Te Ao Māori Is About Wairua and WBIs Cannot Replicate This

When Māori connect, they do so with an energy transfer between 2 people, which conveys many things, including meaning to nuanced words. As such, te ao Māori is about wairua (spirituality) and Mauri (energy or life force), which respondents felt could not be conveyed by a digital tool. The 1D nature of the WBIs often felt empty to participants, robotic, and therefore not healing. For some, there was a perception that turning to WBIs would further drive participants away from te ao Māori and may worsen the underlying illness mechanism. A participant in stated the following:

We need that human connection. But it’s not just physical connection. It’s the frequency, it’s the energy, it’s the Wairua

Group 1

A participant stated the following:

Well-being for Māori comes from connection. I think well-being, particularly in terms of Māori well-being, you know? The energy and frequency that comes with healing...um...is can be misinterpreted; can be absent

Group 2

Another participant stated the following:

The last thing you want is for people to be dependent on digital apps for their well-being. That would be disastrous when people don’t know how to go to other people in a group and don’t be empowered enough to heal themselves

Group 1

#### Māori Models of Health Are Holistic, Whereas WBIs Are Singular in Focus

Many participants reflected on Māori models of healing as being holistic and considering all areas of well-being. Specifically, one was unlikely to become well or heal from illness by just considering 1 facet of treatment (eg, thinking styles), when being well also included good physical health, strong connections to whānau (family) and whenua (the land), whanaungatanga (relationships), a connection to a higher purpose and meaning, and spirituality, all of which were considered essential for people to flourish. The approach of WBIs was largely seen to be singular in focus and did not fit with te ao Māori views of what was needed for healing. It was recognized that the perceived approach of WBIs was not aligned with Māori models of health and was more aligned with traditional Western models of treatment, such as psychological therapy or a medical model.

The common themes demonstrating the desire for a holistic approach included being able to meet the needs of whānau through accessing food, forming relationships, and recognizing that conversations about Mauri and spirituality were absent. Even when WBIs incorporate te reo Māori (the Māori language), the use of kupu (words) are often perceived as tokenistic, with poor translation that fails to capture the nuances of te reo Māori.

#### WBIs Could Be a Strand in the Weaving of a Well-Being Kete

Despite the belief that WBIs were unlikely to meet the ideal delivery of an intervention to support the well-being of Māori, many participants were open to considering using a WBI. Specifically, there was a perception that WBIs could be used as an adjunct to a treatment or therapeutic intervention already underway, particularly when there was already a strong relationship with the therapist or the care team. A participant stated the following:

I feel like I could get the information I need to do more. Cause, obviously, an app can only go so far, and your mental health is really...you know? You’re paddling that waka [boat]...People can help you, but you’re the main navigator of that boat...I think it will help start it will help you build the waka and get it onto the water, um, but then yeah...I am guessing that you would have to put in the work yourself to go find other resources

Interview 3

Another participant stated the following:

Am just not sure we would be able to actually heal people properly, but it could help at least getting them toward the resources they need

Group 3

### WBIs Could Be Helpful in the Right Context

#### Overview

The participants noted several strengths of WBIs and were open to WBIs being a tool that might benefit some people more than others. Context was important, as was the support around the WBI. Specifically, there was a view that while WBIs might be good for some people, using them in isolation, without connection to people, might actually exacerbate issues.

#### WBIs Could Be Useful for People Who “Moved With the Times”

There was a perception that WBIs might suit those that have moved away from traditional modes of healing, such as the younger generations, but those groups did not always support this view. Many participants reported that they felt “older,” meaning they were more likely to struggle with WBIs than younger generations. There was a strong sense that rangatahi (young people) would be more inclined to use WBIs than kāumatua (elders), who were less confident with technology. This was observed in this study by the difficulty many kāumatua experienced accessing the stimulus WBI and the confusion around what the application was asking. The participants often indicated that they did not believe they could learn new technology and tended to avoid using it. As such, the perceived stress of using the WBIs outweighed the potential benefits. A participant stated the following:

I think the young Māori people might have embraced it, but the older ones, we say, “It’s pākehā! [non-Māori].” They won’t; they won’t switch onto it. Could you imagine your mother doing it?

Interview 2

Younger participants tended to voice more concerns about functionality and technological issues with WBIs and less about the values disconnection expressed by the older participants. While open to using WBIs, the younger participants still had reluctance to do so. A low tolerance for poor functionality was indicated, with participants highlighting that the complicated log-in processes or errors would lead to them feeling frustrated and uninstalling an application. This indicated that significant barriers to use were complicated sign-up or sign-in processes and outdated or faulty technology. A participant stated the following:

I feel like I would probably uninstall it. I mean, I don’t really have the patience for broken web pages and things...I don’t think most people do. Especially on phones and your meant to be doing a million things at once

Group 3

The younger participants also supported the views of the older participants around holistic care and the desire for a face-to-face connection. Thus, the assumption that WBIs were embraced by rangatahi was not necessarily true. The younger participants wanted interpersonal connection and did not think WBIs could meet this. WBIs were instead seen as a source of information but unlikely to be considered for therapeutic intervention or connection with others.

#### Apps Could Be a Tool but Not the Solution: a Blended Care Approach Is Needed

Many participants felt that they would be more open to using a WBI when integrated in the context of an existing, trusted relationship. The perception was that the role of the relationship would be to facilitate healing, while the WBI could serve for monitoring, support, providing information, promptly answering questions, and potentially facilitating connections to others beyond the primary the relationship. The relationship would center around wairua, whanaungatanga, and whānau, while the WBI might provide exercises, reminders, and ways to keep track of important things. Relaxation and mindfulness recordings were specifically noted as something that could be provided or used by participants in WBIs. Contacting a trusted support or therapist through the WBI was also seen as a way to increase whanaungatanga and healing if used in a timely and appropriate manner.

### Barriers to Using WBIs for Māori

There were several barriers to using WBIs for participants, and many participants shared that they believed other Māori would also experience some of these challenges.

#### WBIs Come With an Upfront Cost That May Drive Inequitable Access

Having the technology to run WBIs includes an internet connection, an internet-enabled mobile phone or computer, and ongoing data. Cost was a barrier for many participants, who often did not have data on their phones or relied on free Wi-Fi connections in public spaces. Some participants spoke of the challenge of feeding their family and that this would take priority over the financial cost of using a WBI. This was evidenced by the questions around the app’s functionality and if it had features that would enable them to access food for their whānau (family).

#### Technical Issues Put People Off

Technical issues were a frequent complaint and often discouraged people from continuing to use a WBI. This was particularly salient when the difficulties were related to logging into a platform for the first time. Many of the older participants felt confused by log-in demands, and this initially created the perception that they would not be able to use WBI as it was too “technical” for them. The participants felt that the upskilling required to use a WBI would outweigh the benefits, particularly when there was already a belief that WBIs were not a good fit.

#### Literacy and Language May Make Engagement Difficult

At least 1 participant spoke of challenges with reading and writing and said that many of the WBIs available were text heavy and did not cater to people with reading difficulties. Language was particularly an indicator of this, with low use of te reo Māori kupu (words), the selection of only limited imagery of Māori people or images that would resonate with Māori, and a lack of clear use of Māori concepts and frameworks in the tools. When imagery or words were used, they were often inappropriate or superficial. The language and tone used were also considered important, with some older participants identifying that web-based communication was harder for them to understand. Several participants asked if the WBI was available in te reo Māori, as this was a preference. However, there was recognition that many Māori are still regaining te reo Māori; therefore, to be accessible, WBI should offer a range of te reo Māori options, from having key words in te reo Māori to being fully in te reo Māori.

#### Mistrust Due to Years of Systemic Racism and Broken Promises Create Barriers

Several participants reported feeling suspicious of the whakapapa (history and process of coming to be) of WBIs and the research. There were concerns about who was behind the WBI design or who was communicating with participants through the WBI and what their motivation was. The participants appeared to have a lack of confidence that the WBIs would maintain their privacy and instead felt that information may be shared with agencies that might later penalize users.

On a simple level, the participants often did not trust that the person behind the WBI or the web-based therapy was invested in talking to them. There were concerns that the person could be doing other things while engaging in the healing conversation or that the person using the WBIs would not be a priority. A participant stated the following:

I share something you share something you know that kind of kōrero or that I don’t know if they’re listening. I mean, while I’m talking, what are they doing? Emailing somebody?

Group 1

The participants raised further concerns about the confidentiality of information shared with WBIs and how this would be used, particularly with regard to information potentially being shared with government agencies and the potential negative impact this could have on the participant and their whānau. This mistrust was not limited to WBI but to the researchers, the institutions, and the groups that supported it. A participant stated the following:

What puts me off is that there is nothing that comes across as [Māori]. This is a pākehā app for pākehās, pākehās solutions, pākehā reference pākehā, you know? And I think that’s one of the, um, big issues of todays, um, well-being solutions is that pākehā are trying to form solutions using methodologies that aren’t Māori, you know?

Group 1

Many participants reported struggling with interventions that Pākehā developed as they lacked the depth of understanding about the historical impact of colonization on Māori and how this may impact why the person is seeking help. Many felt that interventions and Pākehā therapists and WBIs could not address this as they did not have these lived experiences. A participant stated the following:

I would want someone who can understand what lived experience is...

Group 2

Another participant stated the following:

I prefer to speak with people, real people. And when it comes to like, um, some of my historical stuff, then I would want someone who can understand what lived experience is. You know, email doesn’t cut it either, you know, again who is this person that I’m emailing?

Group 1

Mistrust was further exacerbated by the perception that Māori did not design many WBIs for that were reportedly for Māori. This was not only due to the WBI being perceived as a poor fit for te ao Māori but also due to Pākehā ultimately designing many WBIs for Pākehā (or the dominant paradigm) and then adapting (often poorly) these for Māori. Therefore, very few interventions were specifically designed for Māori, and even fewer were created by Māori. A participant said the following:

There’s no Māori kupu in here. Not even a “kia ora,” welcome

Group 1

Another participant said the following:

They had a real photo, but, um...whoever created didn’t look at the photo properly and it had somebody poking their tongue. So what time was given to that? I felt it was lacking big time cause if I’m not in a great space and I see somebody poking their tongues, saying, you know? I’m feel challenged! What’s up with that?

Group 1

The participants were also wary of research teams that used a Māori interviewer but did not involve Māori in key decision-making. Questions were raised around the motivations of the teams and whether they came from genuine caring and a desire to reduce inequities (it was noted that the interviewer was challenged about this on >1 occasion). There was a belief that those teams that did not have genuine motivation would create solutions that were unlikely to resonate with Māori. A participant stated the following:

You know, they’re not using Māori or mātauranga Māori [knowledge] or Māori methodologies to create Māori solutions or do the research. You know they have got a brown face here, but I mean, who’s behind it and why they behind it? You know?

Group 4

### Ways to Improve WBIs to Help Engagement With Māori

Despite reservations about WBI, the participants provided several recommendations that could make WBIs more engaging for Māori. These were specifically about features of WBIs that would make them more engaging for Māori who may be interested in using WBIs.

#### Māori Imagery Is Key for Māori to Connect

The most common subtheme was that the participants wanted to see themselves reflected in the WBI. Imagery was important, and the participants wanted to see a mix of images, including pictures of Māori people, whānau (families), and hapu (communities). This imagery also included other key things such as bodies of water, native bush, maunga (mountains), Māori art and carvings, and buildings easily identified as Māori (eg, wharenui—meeting houses). This range of imagery tied back to a sense of holistic approaches to well-being, including the connection to people, to whenua (land), and to key important spiritual sites.

The participants also highlighted that Māori are diverse people and that not just 1 image connects with all Māori. Images depicting kapa haka or individuals performing a pukana (facial expression) were frequently viewed as oversimplified and occasionally offensive, simplifying the complexity of Māori culture. Instead, the participants would be drawn to WBIs that used a range of imagery of people showing the diversity of Māori. A participant stated the following:

A diverse range of Māori women. Um...sizes, ages...um, you know, skin tone. You know? Just like everything

Interview 3

This was considered important by almost all participants, particularly as some participants identified that Māori are a strongly oral and visual culture with images of kowhaiwhai (weaving), whakairo (carving), and taonga (treasured items) drawing the eye and creating connection. For participants, many WBIs still mimicked clinical rooms in terms of the colors used and simple pages, which further created a sense of depersonalization and disconnection. A participant stated the following:

It seems very like clinical in a way, um, because of the coloring. Like, it’s all blue, like, it kind of just makes me think of, like, the doctors, um, like, it’s very formal I think

Group 3

#### Māori Models of Well-Being Should Be at the Heart of All Interventions

Several participants noted that if WBIs interventions could be constructed around Māori models of well-being, WBIs would likely be more engaging for Māori. The key model suggested was Te Whare Tapa Whā [45]. While other participants did not explicitly outline a model, they articulated the need for a holistic approach to care that supported all aspects of wellness, including connection to culture as a path for healing. A participant stated the following:

Inclusive of the mental effort, you know the spiritual and all that stuff and the physical but so is this are they talking about holistic well-being or are they just talking about mental well-being because you can’t have one it’s, like, Te Whare Tapu Whā thing, ah, you can’t just focus on the one when you are expecting others to fall into place you have to work on the whole lot

Group 1

Another participant said the following:

There needs to be some sort of cultural, um, tool that helps...that grounds people. I guess whakapapa was one of those things

Group 1

#### Improved Integration of the Te Reo Māori Language Would Make WBIs More Appealing

The participants agreed that correct and appropriate use of te reo Māori was important if WBIs were to engage Māori. Recognizing that the fluency of te reo varied, the participants wanted WBIs to be able to be modified based on the user’s fluency (such as entirely in te reo Māori or with only a few words). Even for those only beginning their te reo journey, keywords such as a greeting should be used meaningfully. The participants also noted that, based on past experiences, simply translating a few words into te reo lost the nuances of the language and could lead to misunderstanding and, at times, felt tokenistic. There was an emphasis on the correct use of Māori words, which often carry contextual meanings. Tokenistic inclusion of these words can lead to misunderstandings or, at worst, be offensive. Therefore, people developing WBIs for Māori needed someone fluent in te reo Māori working on the content rather than using simplified translation tools.

#### By Māori for Māori

A strong theme that came through was that WBIs, interventions, and tools that were developed by Māori for Māori resonated more strongly with Māori participants and were more likely to be engaging and used. Trusted institutions and sites tended to result in resources that were more readily used. A participant said the following:

I have searched through...through Te Ora [website] you know to find out stuff I think because it’s a Māori organization there’s a sense of connection being a Māori organization

Group 1

A clear understanding of the whakapapa (history and origin) of the research project and the research team was deemed crucial, ideally with the project being designed and led by Māori for Māori. In addition, there was discussion about incorporating key models of health and acknowledging the influence of significant Māori figures in the project’s development.

## Discussion

### Principal Findings

This study is one of the first to explore the views of Māori, the indigenous people population of New Zealand, about WBIs. Although participants could see the potential benefits of WBIs when used in the context of a strong existing relationship, there were concerns that a digital tool would not be able to facilitate healing due to the perception of WBIs being 1D in their focus rather than holistic, the potential to drive people further away from te ao Māori, and the lack of genuine connection that could be made on the web. Significant barriers to using WBIs were highlighted, including the impact of social inequities, which hindered access to the technology needed to engage with WBIs. Educational disadvantage also contributed to difficulties, particularly with text-heavy platforms. In addition, concerns about confidentiality and mistrust in the motivations of researchers (and government) due to experiences of colonization were clear.

This study makes a unique contribution to understanding WBIs, how they fit with the view of Māori as the indigenous people of New Zealand, and how indigenous communities may perceive and respond to WBIs. Our findings are in contrast with previous studies that explored uptake and engagement with WBIs in indigenous populations and ethnic minority groups. A recent scoping review exploring the use and uptake of web-based therapeutic interventions among indigenous populations in Australia, New Zealand, the United States, and Canada found moderate uptake of WBIs and potentially improved health outcomes associated with them [36]. Of the 31 studies, 9 (29%) were from New Zealand, with 3 (10%) relating to 1 web-based cognitive behavior therapy–based intervention (Smart, Positive, Active, Realistic, X-factor thoughts), 1 (3%) relating to minimizing risky alcohol use, and others (n=5, 16%) on specific noncommunicable diseases. The review findings reported a more positive experience with WBIs, possibly because of the younger age of participants and the way the WBIs were implemented, for example, delivered in school-based settings or with significant input and coproduction with Māori. Similar to our findings, the review highlighted the importance of tailoring content and presentation formats to ensure cultural relevance; appropriateness; and a customizable, easy-to-use interface. Another systematic review looking at the use of digital technologies to improve the mental health and well-being of indigenous people reported 27 studies that generally support the effectiveness of digital technologies in aiding the provision of mental health services but acknowledged that decolonizing and culturally appropriate approaches are needed [46].

The studies in these reviews generally do not examine the user experience of using the WBIs in depth. The review was limited in the degree to which it could explore barriers to accessing WBIs resulting from cultural and linguistic diversity, low health literacy, limited digital capabilities, and infrastructural and resource limitations for individuals and communities in different geographic locations—concepts that our participants expressed as potential barriers to WBI use. Similarly, a recent rapid review examining the use of web-based care in indigenous populations highlighted several barriers to engagement with web-based care—cost, accessibility, digital literacy, and language [47]—which align with our findings. The review emphasized the importance of building relationships and trust and ensuring the infrastructure is present to support technology navigation with indigenous populations, echoing our findings. Our study builds on these reviews by being one of a few studies to examine the views of Māori using qualitative methodology, which may explain the more in-depth findings articulated by respondents.

### Limitations

Several limitations for this study exist. First, although the sample size was large for a qualitative study, Māori, like all indigenous people, are diverse; therefore, the views reflected in this study may not apply to all Māori. These views can likely be beneficial in shaping the development of WBIs. However, an effective WBI for Māori should be developed by Māori with the understanding that one intervention will not fit everyone. Instead, WBIs need to be developed with Māori models of well-being being central for use alongside a strong kanohi-ki-te-kanohi (face-to-face) therapeutic relationship. Previous reviews [36] recognize that the definitions of health often used are less holistic and relational than indigenous models of health and well-being, which may affect the interpretation of published studies in this area or result in studies appearing to be more effective than what would be perceived from a more holistic framework.

Second, this study explored the views of WBIs and used an existing intervention as an example for participants. This means that interventions that may have a different development process (eg, Māori developed) may have resulted in different views by the participants around the acceptability and usability of WBIs. The views of the participants may also be affected by the stimulus chosen as the example.

Finally, while most of the research team were Māori, the researchers varied in the strength of their connection to te ao Māori and their knowledge of this. This means that the analysis of the transcripts and subsequent findings may have varied if other people with different understandings and connections were to analyze the transcripts.

### Reflections

One challenge for the research team was to avoid replicating some of the concerns and barriers regarding WBIs that were raised by the participants. Specifically, one aspect was the composition of the research team, where the individual conducting participant interviews was Māori, while the senior authors of the paper were not Māori. For context, this project is 1 part of a 2-part study designed from the onset with a diverse team in consultation with a Kaupapa Māori nongovernment organization. MCBP was involved in early discussions with the research team about the project and was brought into the team in recognition of the mātauranga Māori (knowledge unique to Māori) that she brings. MCBP was supported to be an active and equal member of the research, including being provided with support to upskill in qualitative methodologies. PH and HW were engaged in reviewing the themes to ensure interpretation and understanding were correct and to add to the richness of the interpretation, as the research team did not want to assume understanding and acknowledged their limitations around the lived experiences of Māori. Author order was decided among the team (MCBP, HW, PH, LD, and AHYC) based on roles in the project, with the decision to make MCBP and LD joint lead authors in recognition of the different roles in the project. We recognize that Māori should ideally lead projects exploring the view of Māori, and the experience that MCBP has within this project has meant that she has been able to colead a subsequent project and is beginning to develop her own research pathway as a Māori researcher with lived experience.

### Conclusions

Through in-depth qualitative interviews and focus groups with Māori (indigenous people of New Zealand), WBIs were found to be generally considered a poor fit for Māori as the design of WBIs did not align with the Māori worldview or concepts of well-being. This contrasts with previous findings, where WBIs have been shown to be effective in supporting mental health and well-being and can overcome some of the traditional barriers to help seeking. With the large number of WBIs being developed, these findings are important in highlighting key considerations for WBIs to promote engagement with Māori, particularly considering how well WBIs fit with the indigenous worldview and how they meet the needs of indigenous communities in a culturally appropriate manner. While WBIs may have a place in supporting the well-being of Māori, WBIs alone are unlikely to achieve the same benefits expected for a non-Māori population and may further drive health inequities if not properly implemented and supported.

## Abbreviations

WBI: web-based intervention

## Data Availability

The data sets generated and analyzed during this study are not publicly available due to participants not consenting to the sharing of data beyond the research team with consent given for publication.
